# Optimization of Emerging Extraction Techniques for Phenolic Compounds from *Pinus radiata* Bark: Antioxidant, Thermal Stability and Antibacterial Properties

**DOI:** 10.3390/antiox15050565

**Published:** 2026-04-29

**Authors:** Danilo Escobar-Avello, Tomás Oñate-Valdés, Víctor Ferrer, Cecilia Fuentealba, Sergio Benavides-Valenzuela, Gustavo Cabrera-Barjas, Gastón Bravo-Arrepol, Ady Giordano, Beatriz Gullón, Jorge Santos

**Affiliations:** 1 Unidad de Desarrollo Tecnológico, Universidad de Concepción, Coronel 4191996, Chile; tonate@udec.cl (T.O.-V.); v.ferrer@udt.cl (V.F.); c.fuentealba@udt.cl (C.F.); 2 Centro Nacional de Excelencia para la Industria de la Madera (CENAMAD)—ANID BASAL FB210015, Pontificia Universidad Católica de Chile, Santiago 7820436, Chile; 3 Escuela de Nutrición y Dietética, Facultad de Ciencias de la Rehabilitación y Cuidado de Vida, Universidad San Sebastián, Campus Las Tres Pascualas, Lientur 1457, Concepción 4060000, Chile; sergio.benavides@uss.cl (S.B.-V.); gustavo.cabrera@uss.cl (G.C.-B.); 4 Facultad de Ciencias, Universidad San Sebastián, Campus Las Tres Pascualas, Concepción 4060000, Chile; gaston.bravo@uss.cl; 5 Departamento de Química Inorgánica, Escuela de Química, Facultad de Química y de Farmacia, Pontificia Universidad Católica de Chile, Santiago 7820436, Chile; agiordano@uc.cl; 6 Departamento de Enxeñaría Química, Facultade de Ciencias, Universidade de Vigo, 32004 Ourense, Spain; bgullon@uvigo.gal; 7 Instituto de Agroecoloxía e Alimentación (IAA), Universidade de Vigo, Campus Auga, 32004 Ourense, Spain; 8 ARCP—Associação Rede de Competência em Polímeros, 4200-355 Porto, Portugal; jorge.ucha@arcp.pt; 9 LEPABE—Faculty of Engineering, University of Porto, Rua Dr. Roberto Frias, 4200-465 Porto, Portugal; 10 ALiCE—Associate Laboratory in Chemical Engineering, Faculty of Engineering, University of Porto, Rua Dr. Roberto Frias, 4200-465 Porto, Portugal

**Keywords:** plant bark, polyphenols, tannins, green extraction, antioxidant capacity, antimicrobial activity, mass spectrometry, thermal stability, biomaterials, circular bioeconomy

## Abstract

Conventional and emerging extraction methods for recovering phenolic compounds (PCs) from *Pinus radiata* bark were investigated for their potential use in bio-composites and bio-based biomaterial applications. To optimize the recovery process, a Response Surface Methodology (RSM) based on a Box–Behnken design was used to evaluate the effects of extraction time (20–100 min), temperature (20–80 °C), and water or ethanol-water solvent concentrations with *β*-cyclodextrin (*β*CD) or NaOH (0.5–1.5% *w*/*v* CD/db). Polyphenolic profiles of the extracts were characterized using Fourier transform infrared spectroscopy (FTIR), LC-LTQ-Orbitrap-MS, and matrix-assisted laser desorption/ionization time-of-flight mass spectrometry (MALDI-TOF-MS). Thermogravimetric analysis (TGA) and differential scanning calorimetry (DSC) were used to evaluate the thermal stability and degradation behavior of the powdered extracts. Antioxidant capacity (DPPH, FRAP, ABTS) and antibacterial activity against *Escherichia coli* and *Staphylococcus aureus* were assessed by spectrophotometric assays and the agar diffusion method, respectively. Highest extraction yields were obtained using alkaline extraction (14.32%) and ultrasound-assisted extraction (UAE) (13.86%), followed by ethanol extraction (12.74%). Minimum inhibitory concentration (MIC) for P-*β*CD was 0.04 mg/mL, and the minimum bactericidal concentration (MBC) was 0.32 mg/mL against *S. aureus*. These results suggest a strong inhibitory capacity at low concentrations and the potential incorporation of these extracts into bio-based antimicrobial biomaterials.

## 1. Introduction

Forest industries frequently dispose of residues such as bark, branches, foliage, leaves, and sawdust by burning or discarding, leading to both economic and environmental consequences. Currently, an estimated 5 billion tons of biomass waste from the agroforestry and food sectors are produced worldwide each year, accounting for 3.3 billion tons of CO_2_ emissions [1]. However, this biomass can be valorized due to its valuable molecules, which possess antioxidant, antimicrobial, and antifungal properties [2]. In this context, the bark of *P. radiata* is of particular interest. This species is one of the most important for commercial forestry in several countries, including Chile, New Zealand, Australia, and Spain. In Chile alone, *P. radiata* plantations cover more than 1.3 million hectares, representing a major source of lignocellulosic biomass and associated residues [3]. Among these residues, pine bark is particularly valuable due to its high polyphenolic content.

Pine bark is rich in phenolic compounds, including flavonoids, phenolic acids, and condensed tannins, which often exist as glycosylated conjugates [4,5]. Proanthocyanidins (condensed tannins) are particularly abundant and formed by oligomeric and polymeric chains of flavan-3-ol units such as catechin and epicatechin. These compounds have strong antioxidant activity, donating hydrogen atoms or electrons and stabilizing free radicals [6]. In addition to their antioxidant capacity, proanthocyanidins interact with microbial cell membranes and proteins, contributing to antimicrobial activity [7].

Several studies have investigated the extraction of phenolic compounds from pine bark using conventional solvents, such as water, ethanol, and mixtures of water and organic solvents. For instance, extracts from the bark of *P. pinaster*, which is commercially known as Pycnogenol^®^ (Horphag Research, Geneva, Switzerland), have demonstrated strong antioxidant and antimicrobial properties and have been extensively studied for use in nutraceuticals and pharmaceuticals [8,9]. Similarly, extracts from the bark of *P. brutia*, *P. nigra*, and other pine species have shown antibacterial activity against both Gram-positive and Gram-negative bacteria [10,11]. However, the efficiency of phenolic extraction and biological activity depend on the extraction method, solvent polarity, and processing conditions.

In recent years, new methods have been explored to improve the recovery and functionality of phenolic compounds from plant materials. Ultrasound-assisted extraction (UAE) is a popular method because it enhances mass transfer and disrupts plant cell walls, increasing extraction yields and reducing processing time [12]. Alkaline extraction has also been reported to release phenolic compounds from lignocellulosic structures, changing the distribution of tannins and other phenolics in the extract [5]. Another approach uses cyclodextrins, particularly *β*-cyclodextrin, to enhance the solubility, stability, and bioavailability of phenolic compounds [13,14]. In particular, the bark of *P. radiata* has been extracted using both conventional solid–liquid methods and emerging methods, such as ultrasound and microwaves [4,15,16]. However, to our knowledge, the encapsulation or extraction of phenolic compounds from *P. radiata* bark with *β*-cyclodextrin has not been reported.

The characterization of phenolic compounds beyond extraction efficiency is essential for the understanding of the functional properties of bark extracts. Advanced analytical techniques, like Orbitrap-MS and MALDI-TOF, are used to identify flavonoids and proanthocyanidin oligomers in plant extracts [5,17]. These techniques allow for detailed structural characterization of phenolic compounds, including their molecular weight distribution and oligomerization patterns. Thermal analysis techniques like TGA and DSC provide valuable information about the thermal stability and physicochemical behavior of phenolic extracts, relevant for their potential biomaterial applications [4,5]. Thus, understanding how extraction strategies affect the recovery and stability of phenolic compounds is essential for optimizing the valorization of this biomass resource. This study is novel because it directly compares traditional and emerging extraction methods using the same batch of initial pine bark biomass. Our study explores one-step extraction and encapsulation of pine bark phenolic compounds and links extraction methods to the functional performance of the biocomposites.

The aim of this study was to evaluate the influence of different extraction strategies on the chemical composition, thermal properties, and antimicrobial activity of *P. radiata* bark extracts. The results provide valuable information for potential applications in functional biomaterials, especially in the food industry.

## 2. Materials and Methods

### 2.1. Chemicals

All chemicals and reagents were of analytical grade. Gallic acid, Folin–Ciocalteu reagent, ABTS (2,2′-azino-bis(3-ethylbenzothiazoline-6-sulfonic acid)), and TPTZ (2,4,6-tri(2-pyridyl)-S-triazine) were used. DPPH (2,2-diphenyl-1-picrylhydrazyl), sodium acetate trihydrate, potassium persulfate, and iron(III) chloride hexahydrate were obtained from Sigma-Aldrich (St. Louis, MO, USA). Ethanol, sodium hydroxide, sodium carbonate, hydrochloric acid, and acetic acid were obtained from Winkler in Santiago, Chile. Light exposure was avoided when handling the standards.

### 2.2. Raw Material

The *P. radiata* bark, supplied by the Arauco Company in Chile’s Bio-Bio region, was carefully selected to exclude wood and other foreign materials. The bark was then pulverized using an M8 Breuer hammer mill (Breuer, St. Vith, Belgium) and sieved to obtain particle sizes ranging from 0.60 to 4.73 mm. The moisture content was determined by gravimetric analysis using a high-precision BMA H50 thermobalance (BOECO, Hamburg, Germany). A 1 g sample of *P. radiata* bark was heated to 105 °C until a constant weight was achieved, yielding a moisture content of 10.59 ± 0.37%. To ensure accuracy and reproducibility, measurements were carried out in triplicate.

### 2.3. Extraction of Phenolic Compounds

#### 2.3.1. Conventional Extractions

Conventional water and ethanol extractions were performed in 50 mL tubes. The tubes were sealed and placed in glass flasks, which were then heated. To prevent degradation of the phenolic compounds due to light exposure and solvent evaporation, the glass flasks were covered with aluminum foil during extraction. The effects of different extraction times (20–100 min) and temperatures (20–80 °C) on the process were examined. Then, 2.75 g of *P. radiata* bark was added to the water or the 80:20 ethanol-to-water mixture, maintaining a solid-to-liquid ratio of 1:10 (*w*/*w*). After extraction, the extracts were filtered under a vacuum pump (GM 0.33II, Tianjin, China) using Whatman No. 1 filter paper. To ensure maximum clarity and removal of fine particles, the filtrate was centrifuged at 4000 rpm for 5 min. The resulting extract was stored in an amber vial in the dark at −20 °C.

#### 2.3.2. Ultrasound-Assisted Extraction

Ultrasonic extractions were performed in 50 mL sealed vessels with either distilled water (UAE-W) or an 80:20 (*v*/*v*) ethanol-to-water mixture (UAE-E), using a 1:10 (*w*/*w*) solid-to-liquid ratio, as previously outlined. A GT SONIC P6 ultrasound bath (GT SONIC, Shenzhen, China), operating at a fixed frequency of 40 kHz and 150 W with an ultrasound intensity of 0.25 W/cm^2^, was used to extract 2.75 g of bark samples. Extraction conditions were systematically adjusted with different periods (5 to 25 min) and temperatures (20 to 80 °C). After extraction, the samples were filtered, centrifuged, and stored as described above.

#### 2.3.3. Alkali-Assisted Extraction

Alkali extractions were performed in 50 mL containers with distilled water as the solvent on a heating plate (VELP Scientifica Srl, Monza, Italy). The investigated parameters were extraction time (20–100 min), extraction temperature (20–80 °C), and alkali concentration (0.5–1.5% *w*/*v* NaOH/db) in the solvent, as described by Santos et al. [5]. After extraction, the samples were filtered, centrifuged, and stored.

#### 2.3.4. *β*-cyclodextrin-Assisted Extraction

Extractions were performed using *β*-cyclodextrin (*β*CD), of pharmaceutical grade (99.6%, Haihang Industry, Jinan, China), in 50 mL hermetically sealed vessels in distilled water, which were placed in beakers on a hot plate (VELP Scientifica Srl, Usmate Velate, MB, Italy). The following parameters were systematically investigated: extraction time (20–100 min), extraction temperature (20–80 °C), and *β*CD concentration (0.5–1.5% *w*/*v β*CD/db). For characterization purposes, the extracts were hydrolyzed using a specific method. For this, 0.1% *v*/*v* formic acid was added to each extract sample. After hydrolysis, the samples were centrifuged at 4000 rpm for five minutes. The resulting extracts were then used for characterization.

### 2.4. Experimental Design and Optimization (RSM)

The extraction parameters were optimized using Design-Expert software (version 22.06) and response surface methodology (RSM), employing face-centered central composite designs. For alkali-assisted and *β*-cyclodextrin-assisted extractions, a three-variable design was used (*n* = 17), including NaOH and *β*CD (% *w*/*v*), extraction time (min), and temperature (°C), with five replicates at the central point. In contrast, conventional extraction and ultrasound-assisted extraction (UAE) were optimized using a two-variable design (*n* = 13), with extraction time (min) and temperature (°C) as factors, each with five replicates at the central point. Table 1 provides a detailed overview of the study’s variables and responses.

The selected response variables were maximized using a multi-response surface optimization method. Selection criteria were based on extracts with high yields of phenolic compounds and antioxidant activity, as determined by DPPH, ABTS, and FRAP methods. Optimal extraction conditions were determined using the Design Expert software (version 22.06) and the Desirability Function. Model validation was performed by conducting experiments under the optimal extraction conditions. Then, the values predicted by each model were compared with the experimental data.

### 2.5. Chemical Characterization of the Extracts

#### 2.5.1. Extraction Yield

The extraction yield was determined by measuring the mass of soluble solids remaining after extraction. 2 mL of filtered extract was placed in capsules and heated in a forced-air oven (BIOBASE, Jinan, China) at 105 °C for 16 h. Then, the capsules were weighed, and the percentage of solids in the extract was calculated [3]. All samples were tested in triplicate, and the results were expressed as a percentage of the extracted mass relative to the initial dry mass of the treated bark.

#### 2.5.2. Determination of Total Phenolic Content (TPC)

The Folin–Ciocalteu (F–C) spectrophotometric method was adapted to a 96-well microplate format to determine total phenolic content (TPC) [18]. Briefly, 24 μL of the sample was mixed with 184 μL of Milli-Q water. Then, 12 μL of F–C reagent (2 N) and 30 μL of a 20% sodium carbonate solution were added. Prior to analysis, *P. radiata* bark extracts were diluted 1:100 (*v*/*v*) with Milli-Q water. The reaction mixtures were incubated for 60 min at room temperature (20 °C ± 2 °C) in the dark. Absorbance was measured at 750 nm using a microplate reader (SPECTROstar Nano, BMG Labtech, Ortenberg, Germany). A calibration curve was prepared using gallic acid (10–200 mg/L). The results were expressed as milligrams of gallic acid equivalent per 100 g of dry bark (mg GAE/100 g db). All measurements were performed in triplicate.

#### 2.5.3. Antioxidant Assays

The free radical scavenging activity was evaluated using the 2,2-diphenyl-1-picrylhydrazyl (DPPH) assay. A 0.1 mM DPPH solution was prepared in HPLC-grade ethanol. Briefly, 25 μL of the sample was mixed with 250 μL of DPPH solution in a 96-well microplate, corresponding to a 1:10 (*v*/*v*) ratio. The mixtures were shaken and incubated for 60 min at room temperature (20 °C ± 2 °C) in the dark. Absorbance was measured at 515 nm using a microplate reader (SPECTROstar Nano, BMG Labtech, Ortenberg, Germany). A calibration curve was constructed using gallic acid (1–40 mg/L). The antioxidant activity was expressed as milligrams of gallic acid equivalents per 100 g of dry bark (mg GAE/100 g db). All assays were performed in triplicate.

The FRAP assay was adapted to a 96-well microplate format according to the protocol described by Sricharoen et al. [19], with minor modifications. A 300 mM acetate buffer solution (pH 3.6) was prepared using sodium acetate and adjusted with acetic acid. A 10 mM TPTZ (2,4,6-tris(2-pyridyl)-s-triazine) solution was prepared in 40 mM HCl. Additionally, a 20 mM FeCl_3_ solution was prepared. The FRAP working reagent was freshly prepared by mixing the acetate buffer, TPTZ, and FeCl_3_ in a 10:1:1 (*v*/*v*/*v*) ratio. For the assay, 250 μL of the FRAP reagent was mixed with 25 μL of the sample or standard solution in a 96-well microplate. After shaking, the reaction mixture was incubated at 37 °C for 6 min. A calibration curve was constructed using gallic acid (1–200 mg/L). Antioxidant activity was expressed as milligrams of gallic acid equivalents per 100 g of dry bark (mg GAE/100 g db). All assays were performed in triplicate.

The ABTS radical scavenging activity was determined using the method described by Riquelme et al. [20] with minor modifications. First, a 7.4 mM ABTS solution was mixed with 2.6 mM potassium persulfate (K_2_S_2_O_8_) at a 1:1 volume-to-volume (*v*/*v*) ratio to generate the ABTS radical cation (ABTS^•+^), with the mixture adjusted to pH 7.4. The mixture was left in the dark at room temperature (20 °C ± 2 °C) for 12–16 h before use. Prior to analysis, the ABTS^•+^ solution was diluted with ethanol to achieve an absorbance of 0.70 ± 0.02 at 734 nm. For the assay, 225 μL of the diluted ABTS^•+^ solution was mixed with 25 μL of the sample or standard solution in a 96-well microplate. The reaction mixture was incubated at room temperature (20 °C ± 2 °C) for 30 min. The absorbance was then measured at 734 nm. A calibration curve was prepared using gallic acid (1–20 mg/L). Antioxidant activity was expressed as milligrams of gallic acid equivalents per 100 g of dry bark (mg GAE/100 g db). All assays were performed in triplicate.

### 2.6. Phenolic Profile Characterization

#### 2.6.1. Fourier Transform Infrared Spectroscopy (FTIR) Assay

FTIR spectra were recorded using a Bruker Alpha II FTIR spectrometer (Bruker Optik GmbH, Ettlingen, Germany), equipped with a diamond attenuated total reflectance (ATR) accessory. Thirty-two scans were collected over the 4000–500 cm^−1^ spectral range at a resolution of 4 cm^−1^ [5]. Spectral acquisition and processing were performed using OPUS 7.0 software (Bruker Optik GmbH, Germany).

#### 2.6.2. Matrix-Assisted Laser Desorption/Ionisation-Time of Flight Mass Spectrometry (MALDI-TOF-MS)

MALDI-TOF-MS analyses were performed using an Ultraflex workstation (Bruker Daltonics, Bremen, Germany) equipped with a 337 nm nitrogen laser. Measurements were performed in positive ion mode. After a 150 ns delay, the ions were accelerated to 25 kV. Data were collected from 100 laser shots using the lowest necessary laser energy to obtain sufficient spectral intensity. The mass spectrometer was calibrated using xylooligosaccharides and arabinooligosaccharides (DP 2–6) from Megazyme. For sample preparation, 1 μL of sample solution (1 mg/mL) was mixed with 1 μL of matrix, deposited onto the MALDI target plate, and dried under a stream of warm air. The matrix solution was prepared by dissolving 10 mg of 2,5-dihydroxybenzoic acid (Bruker Daltonics, Bremen, Germany) in a mixture of 700 μL of water and 300 μL of acetonitrile.

#### 2.6.3. Phenolic Profile by LC–LTQ–Orbitrap–MS

Liquid chromatography (LC) analysis was performed using an Accela Chromatographic System (Thermo Fisher Scientific, Hemel Hempstead, UK) equipped with a diode array detector (DAD) and a thermostated autosampler. Chromatographic separation was achieved using an Atlantis T3 column (2.1 × 100 mm, 3 µm; Waters Corporation, Milford, MA, USA). The mobile phase consisted of (A) water containing 0.1% formic acid and (B) methanol. The gradient program was as follows: 0 min: 2% B; 0–2 min: 8% B; 2–12 min: 20% B; 12–13 min: 30% B; 13–17 min: 100% B; 17–18 min: 2% B. This was followed by 5 min of re-equilibration to initial conditions. The flow rate was 0.35 mL/min, and the injection volume was 5 µL, following previous studies [18].

The LC system was coupled to an LTQ-Orbitrap XL mass spectrometer (Thermo Fisher Scientific, Hemel Hempstead, UK), equipped with an electrospray ionization (ESI) source operating in FTMS in negative ion mode. Instrument control and data acquisition were performed using Xcalibur 3.0 software. The mass range was set from *m*/*z* 120 to 2000. Data-dependent acquisition was employed, selecting the most intense precursor ions for fragmentation. The parent ions were fragmented using high-energy collision-induced dissociation (HCD) with a normalized collision energy of 35% and an activation time of 20 ms. The following parameters were applied: sheath gas (N_2_), 50 arbitrary units (a.u.); auxiliary gas, 10 a.u.; sweep gas, 1 a.u.; and capillary temperature, 350 °C. Full-scan MS spectra were acquired at a resolving power of 60,000 (full width at half maximum [FWHM] at *m*/*z* 400), and MS^2^ spectra were acquired at a resolving power of 15,000. Mass accuracy was maintained below 5 ppm, and compound identification was based on accurate mass measurements and MS^2^ fragmentation patterns compared with literature data [4,18].

### 2.7. Thermal Characterization

#### 2.7.1. Thermogravimetric Analysis (TGA) Characterization

For thermal analysis, the liquid extracts were concentrated under vacuum and lyophilized using a Freeze Dry System (LABCONCO, Kansas, MO, USA) at −50 °C and 0.05 mbar for 48 h to obtain a fine powder. Thermogravimetric analysis of the powdered extracts was performed using a TG 209 F3 Tarsus thermobalance (NETZSCH-Gerätebau GmbH, Selb, Germany). Approximately 2–5 mg of the sample was placed in an aluminum crucible and heated in a nitrogen atmosphere (99.995%, AGA) from 25 to 600 °C at 10 °C/min. The TGA curves were recorded to determine the percentage weight loss as a function of temperature, and the first-derivative thermogravimetric (DTG) curves were obtained to evaluate the degradation behavior.

#### 2.7.2. Differential Scanning Calorimetry (DSC) Analysis

Differential scanning calorimetry (DSC) analysis was performed using a DSC 204 F1 Phoenix instrument (NETZSCH-Gerätebau GmbH, Selb, Germany). Approximately 1–2 mg of sample was placed in an aluminum pan with a pierced lid and heated from 25 to 300 °C at a rate of 10 °C/min under nitrogen flow (20 mL/min).

### 2.8. Measurement of Antimicrobial Activity

The antibacterial activities of *Escherichia coli (*ATCC 25922) and *Staphylococcus aureus* (ATCC 6538) were initially assessed using the agar diffusion method. Approximately 25 μL of each extract was applied into wells made in trypticase soy agar (TSA; Merck, Darmstadt, Germany) plates previously inoculated with bacterial suspensions (0.1 mL; 10^5^–10^6^ CFU/mL). The plates were incubated at 37 ± 1 °C for 24 h, and inhibition halos were measured.

Extracts showing antimicrobial activity were further evaluated to determine their Minimum Inhibitory Concentration (MIC) and Minimum Bactericidal Concentration (MBC). The macrodilution technique was performed in Trypticase Soy Broth (TSB; Merck, Darmstadt, Germany), as indicated by the Clinical and Laboratory Standards Institute of the United States of America [21]. A calibration curve was prepared using serial dilutions of *E. coli* and *S. aureus* cultures. Bacterial growth was monitored by measuring absorbance at 600 nm using a UV–Vis spectrophotometer (Hanon, Model i3, Jinan, Shandong, China).

Extracts were serially diluted (1:2), starting from a stock dispersion of 5 mg in 10 mL of distilled water. Each test tube contained 1800 µL of sterile TSB, 100 µL of bacterial inoculum (10^6^ CFU/mL), and 100 µL of the corresponding extract dilution. Tubes were incubated at 37 ± 1 °C for 24 h, and absorbance was measured at 600 nm. Sterile TSB was used as a blank, and inoculated TSB without extract served as the positive control. The MIC was defined as the lowest extract concentration showing no visible growth compared to the positive control. For MBC determination, aliquots from tubes showing no visible growth were plated on TSA and incubated to confirm the absence of colony formation.

### 2.9. Statistical Analysis

All experiments were conducted in triplicate, and results are expressed as mean ± standard deviation. A one-way ANOVA was used to assess group differences, followed by a Tukey post hoc test for multiple comparisons. Differences were considered significant at *p* < 0.05. Analyses were performed using OriginPro 2021 (OriginLab Corporation, Northampton, MA, USA).

## 3. Results and Discussion

### 3.1. Optimization of Extraction of Phenolic Compounds

#### 3.1.1. Extraction Yield (EY)

**Figure 1:**
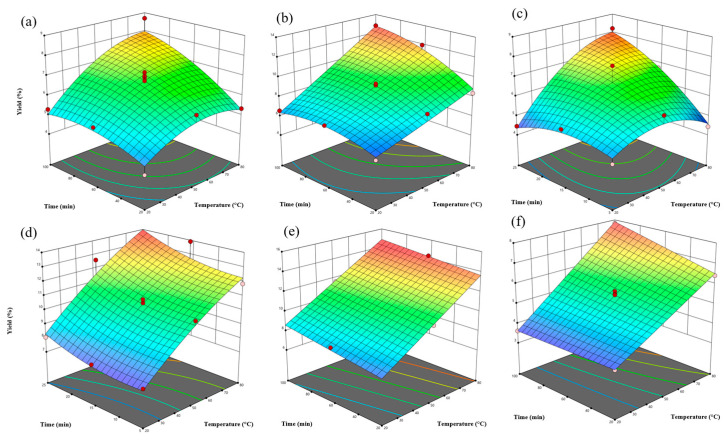
Response Surface for Extraction Yield. (**a**) Water: conventional water extraction; (**b**) Ethanol: conventional 80% (*v*/*v*) ethanol extraction; (**c**) UAE Water: ultrasound-assisted water extraction; (**d**) UAE Ethanol: ultrasound-assisted ethanol extraction; (**e**) Alkali: NaOH-assisted extraction; (**f**) *β*-cyclodextrin: *β*CD-assisted extraction.

Figure 1 shows the response surface plots for extraction yield (EY). The highest yields were obtained at 80 °C and 100 min for conventional water, ethanol, alkaline, and *β*CD extractions. For ultrasonic-assisted extraction (UAE-W and UAE-E), the optimal extraction time was reduced to 25 min. In the studied range, increasing temperature consistently enhanced the extraction yield. The positive influence of temperature is associated with improved solvent diffusivity, reduced viscosity, and enhanced mass transfer [22,23]. The experimental design was limited to 80 °C to prevent thermal degradation of phenolic compounds and maintain system stability at ambient pressure.

The ANOVA results indicated that temperature and extraction time were statistically significant factors (*p* < 0.05) for most extraction methods (see Table S1). However, in the case of UAE-E, the extraction time was not statistically significant (*p* > 0.05). This may be due to the short extraction interval evaluated (5–25 min), during which equilibrium may have rapidly been reached under cavitation-assisted conditions. Similar observations were reported by Ghitescu et al. [24], who found extraction time to be non-significant in the UAE of spruce wood.

*β*-cyclodextrin (*β*CD) concentration did not significantly affect extraction yield, whereas sodium hydroxide concentration had a significant effect (*p* < 0.05), with optimal performance at 1.5% (*w*/*w*). The enhanced yield under alkaline conditions is due to the partial disruption of lignocellulosic structures, including the cleavage of ester bonds and cell wall swelling, which facilitate the release of bound extractives. An earlier study reported that alkaline bark extraction significantly increased the yield of bark from six different pine species extracted with hot water [25].

**Figure 2:**
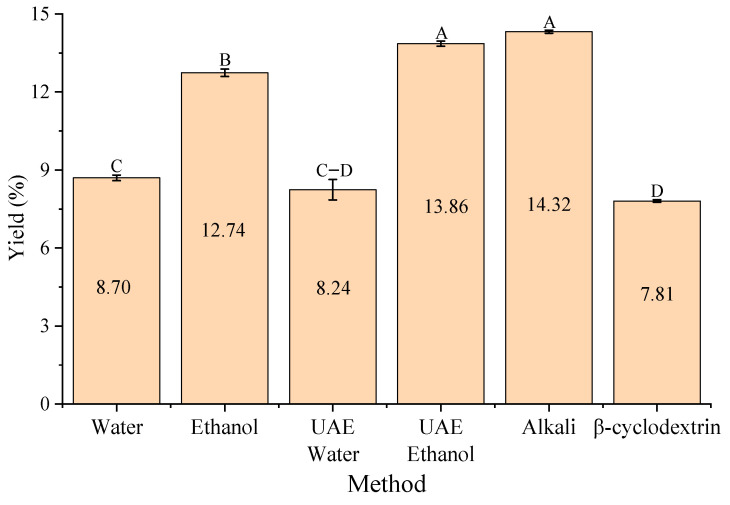
Extraction yield (%) *of P. radiata* bark using different methods. Values are expressed as mean ± standard error (n = 3). Capital letters indicate significant differences between treatments (One-Way ANOVA, Tukey’s test, *p* < 0.05). Water: conventional water extraction; Ethanol: conventional 80% (*v*/*v*) ethanol extraction; UAE Water: ultrasound-assisted water extraction; UAE Ethanol: ultrasound-assisted ethanol extraction; Alkali: NaOH-assisted extraction; *β*-cyclodextrin: *β*CD-assisted extraction.

Figure 2 summarizes the maximum yields obtained using each extraction method. Alkaline extraction (14.32%) and UAE-E (13.86%) produced the highest yields, followed by ethanol extraction (12.74%). Water (8.70%), UAE-W (8.24%), and *β*CD (7.81%) exhibited significantly lower yields (*p* < 0.05).

The superior yield observed with alkaline extraction is consistent with the chemical action of NaOH on the structural components of *P. radiata* bark, which enhances extractability. The yield obtained (14.32%) closely matches our previous pilot-scale study (14.30%) using 1% (*w*/*w*) NaOH in a 750 L reactor [5], supporting both reproducibility and scalability of the process.

Ethanol extraction improved yield compared to water, likely due to its intermediate polarity, which enhances the solubility of semi-polar phenolic constituents in pine bark. The yield obtained in this study (12.74%) is slightly higher than the yield reported in another study using *Pinus elliottii* bark (10.50%) with 80% ethanol at 75 °C, a solvent-to-solid ratio of 1:20, and two hours of extraction [26].

Ultrasound application increased yield in ethanol compared to conventional ethanol extraction, suggesting synergistic effects between solvent polarity and cavitation-induced cell disruption. However, this effect was not observed for water extraction, where UAE-W produced similar yields to conventional water extraction. In contrast, *β*CD extraction may influence phenolic compound recovery [27,28], although not the overall yield. The selective extraction of phenolic compounds through inclusion complex formation between *β*CD and aromatic compounds may explain improvements in phenolic recovery without increasing total extraction yield [13].

#### 3.1.2. Total Phenolic Content (TPC)

**Figure 3:**
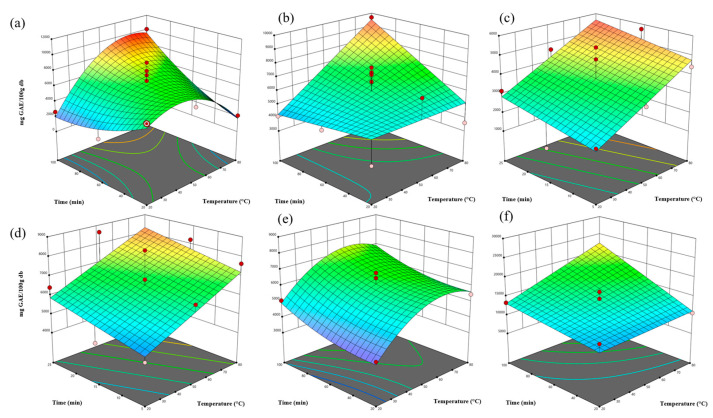
Response Surface for Total Phenolic Content of *P. radiata* bark extracts: (**a**) Water: conventional water extraction; (**b**) Ethanol: conventional 80% (*v*/*v*) ethanol extraction; (**c**) UAE Water: ultrasound-assisted water extraction; (**d**) UAE Ethanol: ultrasound-assisted ethanol extraction; (**e**) Alkali: NaOH-assisted extraction; (**f**) *β*-cyclodextrin: *β*CD-assisted extraction.

The total phenolic content (TPC) of *P. radiata* bark extracts was determined using the Folin–Ciocalteu method. Figure 3 shows the response surface plots obtained using Design-Expert software.

Extraction time and temperature generally increased TPC in most extraction methods. However, TPC decreased to the highest evaluated temperature (80 °C) for conventional water and alkaline extraction. This decline may be due to the thermal degradation, oxidation, or condensation of phenolic compounds such as catechin, epicatechin, and phenolic acids when subjected to prolonged heating [29]. In alkaline media, phenolate ions are susceptible to oxidative reactions, resulting in a loss of the reduced capacity of these compounds [30]. Ethanol and *β*CD systems may stabilize phenolic structures more effectively, thereby reducing the effects of thermal degradation [13,31].

ANOVA analysis revealed that sodium hydroxide concentration significantly affected total phenolic content (TPC) (*p* < 0.05), with optimal performance observed at 1.5% (*w*/*w*). Alkaline treatment enhances the release of bound polyphenols, also known as non-extractable polyphenols (NEPPs), by cleaving ester and ether linkages within the lignocellulosic matrix, thereby increasing the measurable phenolic content [32]. However, excessively high alkaline conditions may promote the oxidative degradation, structural rearrangement, or condensation of phenolic compounds, particularly at elevated temperatures, as explained above.

**Figure 4:**
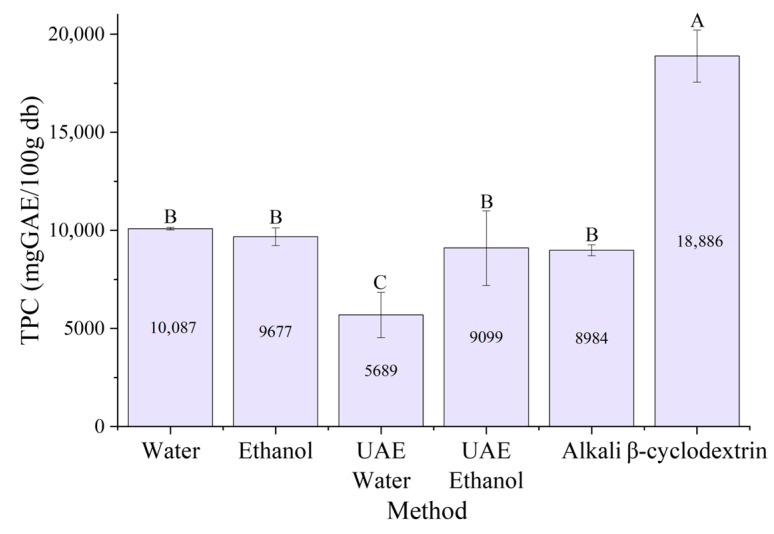
Maximum TPC for each extraction method of *P. radiata* bark. Values are expressed as mean ± standard error (*n* = 3). Capital letters indicate significant differences between treatments (One-Way ANOVA, Tukey’s test, *p* < 0.05). Water: conventional water extraction; Ethanol: conventional 80% (*v*/*v*) ethanol extraction; UAE Water: ultrasound-assisted water extraction; UAE Ethanol: ultrasound-assisted ethanol extraction; Alkali: NaOH-assisted extraction; *β*-cyclodextrin: *β*CD-assisted extraction.

Interestingly, although *β*CD concentration was not statistically significant (*p* > 0.05), *β*CD extraction achieved the highest TPC values. This suggests that *β*CD primarily enhances phenolic recovery through host–guest inclusion complex formation, which improves solubility and stabilizes phenolic compounds during extraction rather than through concentration-dependent effects. Figure 4 summarizes the maximum TPC values obtained for each extraction method.

The highest total phenolic content (TPC), 18,866 mg GAE/100 g db, was obtained using *β*-cyclodextrin extraction. This method produced results that were statistically different from those of the others. However, the water, ethanol, ultrasound-assisted (UAE), and alkaline extraction methods did not differ significantly from each other. Interestingly, alkaline extraction maximizes overall yield, while *β*-cyclodextrin extraction maximizes phenolic concentration. These results highlight the trade-off between the quantity and quality of extraction.

#### 3.1.3. Antioxidant Capacity

**Figure 5:**
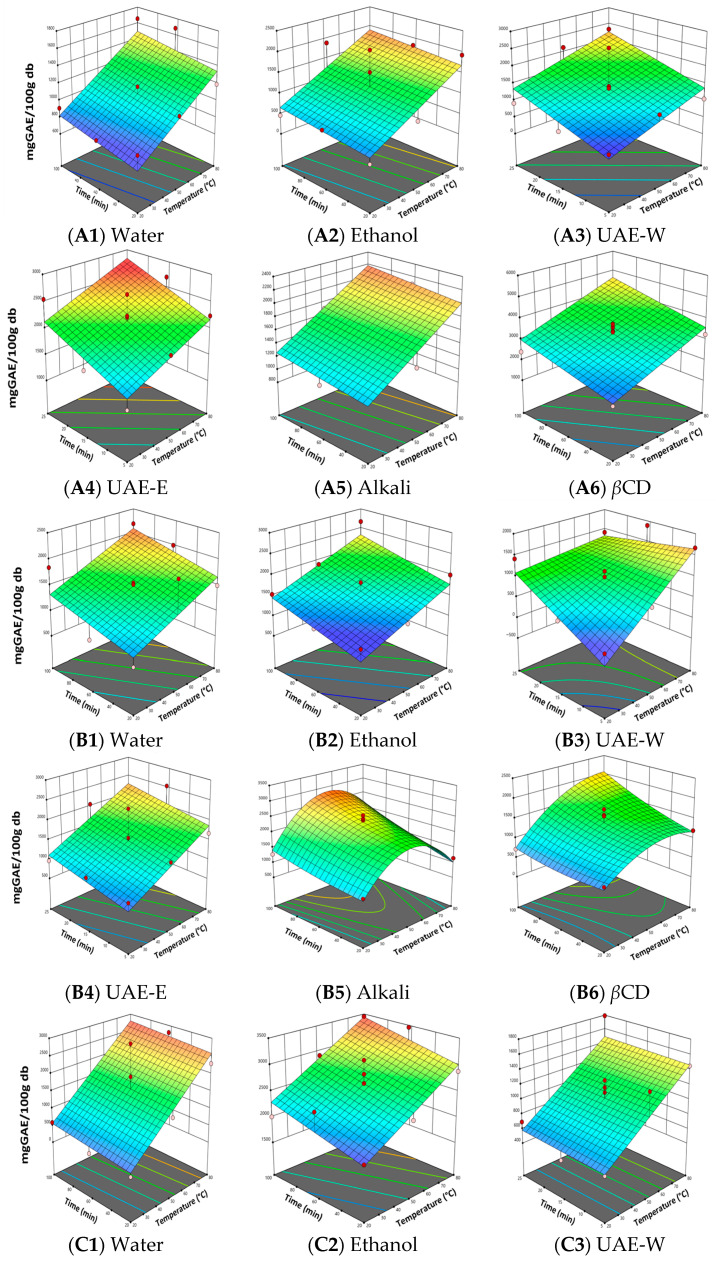

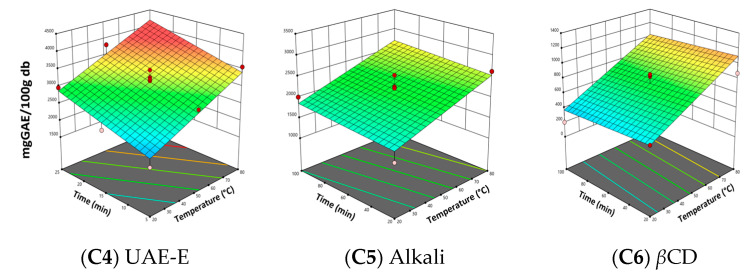
Response surfaces for antioxidant capacity of *P. radiata* bark extracts: FRAP (**A1**–**A6**); DPPH (**B1**–**B6**); ABTS (**C1**–**C6**): (1) Water: conventional water extraction; (2) Ethanol: conventional 80% ethanol extraction; (3) UAE-W: ultrasound-assisted water extraction; (4) UAE-E: ultrasound-assisted ethanol extraction; (5) Alkali: NaOH-assisted extraction; and (6) *β*-CD: *β*-cyclodextrin-assisted extraction. Values are expressed as milligrams of gallic acid equivalent (mg GAE)/100 g of dry basis (db).

The antioxidant capacity of *P. radiata* bark extracts was evaluated using the FRAP, ABTS, and DPPH assays. The experimental data were analyzed using Design-Expert software, and the results are presented as response surface plots (Figure 5).

Temperature had a statistically significant effect on most models, whereas extraction time was generally insignificant (Table S1). Sodium hydroxide concentration significantly impacted antioxidant capacity in both the ABTS (*p* < 0.05) and DPPH (*p* < 0.05) assays, though not in the FRAP assay (*p* > 0.05). In contrast, *β*-cyclodextrin (*β*CD) concentration had no statistically significant effect in any of the assays.

• Maximum antioxidant capacity—FRAP assay

**Figure 6:**
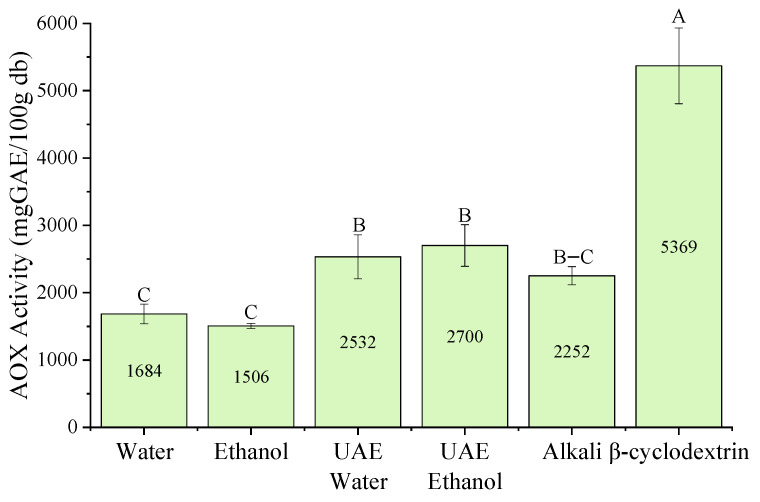
Maximum antioxidant capacity determined by the FRAP assay for each extraction method of *P. radiata* bark. Values are expressed as mean ± standard error (*n* = 3). Capital letters indicate significant differences between treatments (One-Way ANOVA, Tukey’s test, *p* < 0.05). Water: conventional water extraction; Ethanol: conventional 80% (*v*/*v*) ethanol extraction; UAE Water: ultrasound-assisted water extraction; UAE Ethanol: ultrasound-assisted ethanol extraction; Alkali: NaOH-assisted extraction; *β*-cyclodextrin: *β*CD-assisted extraction.

The highest antioxidant capacity (5369 mg GAE/100 g db) was obtained using *β*CD extraction in the FRAP assay (Figure 6), showing a similar trend to that observed for total phenolic content (TPC). Furthermore, both ultrasound-assisted extraction methods (UAE-W and UAE-E) exhibited significantly higher antioxidant capacity than their conventional counterparts, suggesting that ultrasound enhances the recovery of redox-active compounds.

• M aximum antioxidant capacity—ABTS assay

**Figure 7:**
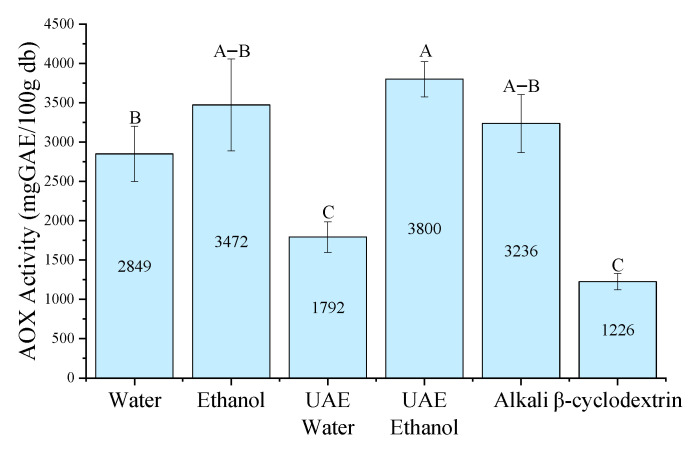
Maximum antioxidant capacity determined by the ABTS assay for each extraction method of *P. radiata* bark. Values are expressed as mean ± standard error (*n* = 3). Capital letters indicate significant differences between treatments (One-Way ANOVA, Tukey’s test, *p* < 0.05). Water: conventional water extraction; Ethanol: conventional 80% (*v*/*v*) ethanol extraction; UAE Water: ultrasound-assisted water extraction; UAE Ethanol: ultrasound-assisted ethanol extraction; Alkali: NaOH-assisted extraction; *β*-cyclodextrin: *β*CD-assisted extraction.

The ABTS assay showed that UAE-E extraction yielded the highest antioxidant capacity, followed by ethanol extraction and alkaline extraction. There were no statistically significant differences between these three methods. UAE-W and *β*CD extraction showed the lowest ABTS antioxidant capacities (Figure 7).

Interestingly, this trend contrasts with the TPC results, which showed the highest phenolic concentration in *β*CD extraction. This discrepancy may be due to potential interactions between the ABTS^+^ radical and the hydrophobic cavity of *β*CD. It has been reported that ABTS^+^ can form inclusion complexes with *β*CD to some extent, which could affect radical accessibility and antioxidant capacity in such systems [33].

• Maximum antioxidant capacity—DPPH assay  

**Figure 8:**
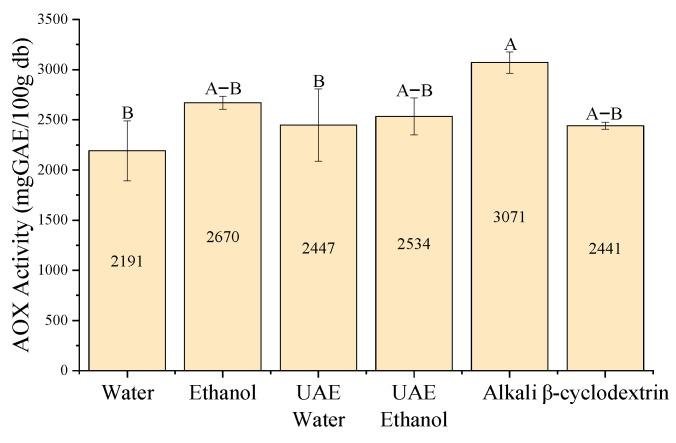
Maximum antioxidant capacity determined by the DPPH assay for each extraction method of *P. radiata* bark. Values are expressed as mean ± standard error (*n* = 3). Capital letters indicate significant differences between treatments (One-Way ANOVA, Tukey’s test, *p* < 0.05). Water: conventional water extraction; Ethanol: conventional 80% (*v*/*v*) ethanol extraction; UAE Water: ultrasound-assisted water extraction; UAE Ethanol: ultrasound-assisted ethanol extraction; Alkali: NaOH-assisted extraction; *β*-cyclodextrin: *β*CD-assisted extraction.

The DPPH assay revealed that alkaline, ethanol, UAE-E and *β*CD extractions exhibited the greatest antioxidant capacity, with no statistically significant differences between them. By contrast, water and UAE-W extractions showed the lowest antioxidant capacity in the DPPH assay (Figure 8).

Overall, the antioxidant capacity generally followed the trend observed for total phenolic content (TPC), although differences depending on the assay were evident. These variations are attributed to distinct mechanisms; while FRAP is strictly based on Single Electron Transfer (SET), ABTS and DPPH operate through both SET and Hydrogen Atom Transfer (HAT) pathways [34]. The lower response in some systems under acidic conditions, FRAP versus neutral conditions ABTS, suggests that the pH-dependent deprotonation of phenolic hydroxyl groups is critical for their reactivity. This highlights the impact of extraction system composition and methodological differences on antioxidant measurements.

#### 3.1.4. Optimization and Comparison of Extraction Conditions and Model Validation

The results obtained from the FRAP, ABTS and DPPH antioxidant capacity assays generally aligned with the observed trends for total phenolic content. However, variations due to assay-dependent differences in the underlying reaction mechanisms and potential interactions between phenolic compounds and the extraction systems were evident. These findings suggest that the recovery of phenolic compounds and the antioxidant potential of *P. radiata* bark extracts are both influenced by extraction conditions.

Based on the response surface models generated using Design-Expert software, the optimal extraction conditions for each system were determined. The predictive capability and robustness of the models were subsequently evaluated through experimental validation performed under the predicted optimal conditions. This approach enabled direct comparison of predicted and experimentally obtained values, confirming the models’ reliability in describing the extraction behavior and antioxidant responses of *P. radiata* bark extracts. Table 2 summarizes the optimized conditions for each extraction system and highlights its principal advantages.

### 3.2. Characterization of the Extract Obtained Under Optimal Conditions

#### 3.2.1. Fourier Transform Infrared Spectroscopy (FTIR)

**Figure 9:**
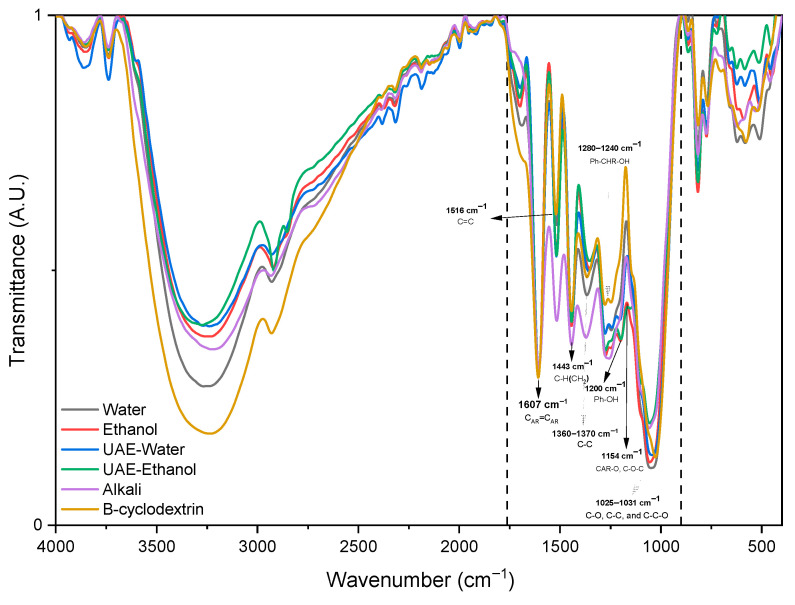
ATR-FTIR spectra of *P. radiata* bark extracts obtained using different extraction systems. The dashed lines show the spectral region where the phenolic bands are located.

The chemical composition of the different extracts obtained from *P. radiata* bark was analyzed using ATR-FTIR spectroscopy. Figure 9 shows the spectra obtained from water, ethanol, alkaline, and ultrasound-assisted (UAE) extractions, and the *β*-cyclodextrin ones. Peak intensities were normalized relative to the aromatic polyphenol ring vibration at 1600 cm^−1^, which served as the internal reference band. Band assignments were based on previous studies of phenolic compounds and tannin-rich plant extracts [5].

The main phenolic compounds found in *P. radiata* bark are condensed tannins, also known as proanthocyanidins. In FTIR spectra, these compounds are associated with three intense absorption bands: 1607 cm^−1^, 1516 cm^−1^, and 1443 cm^−1^. These bands correspond to aromatic C=C stretching vibrations, asymmetric aromatic C=C stretching, and C–H bending of CH_2_ groups, respectively. These bands are characteristic of the aromatic structure of condensed tannins and other phenolic compounds commonly found in pine bark extracts.

In the alkaline extract, a noticeable increase in the intensity of the 1516 cm^−1^ band was observed, which may be associated with partial lignin solubilization or structural modifications induced by alkaline hydrolysis [35]. Additionally, the reduced intensity of the 1155 and 1035 cm^−1^ bands, which are typically attributed to C–O–C and C–O vibrations in oligomeric phenolic structures, suggests the possible depolymerization or degradation of condensed tannins under alkaline conditions. The alkaline extract exhibited a weak absorption band at 1728 cm^−1^, which corresponds to C=O stretching vibrations in unconjugated carbonyl groups. This signal is likely associated with ester hydrolysis reactions that occur in alkaline media and can affect hydrolysable tannins, hydroxycinnamic acid esters, and other phenolic derivatives. However, the low intensity of this band suggests that these structures are present in limited amounts in the extract.

Conventional water extraction, ethanol extraction, and ultrasound-assisted extraction (UAE) showed differences mainly in the fingerprint region associated with condensed tannins. Specifically, bands around 1154 cm^−1^ (C–O stretching in oligomeric phenolic structures) and 1041 cm^−1^ (C–O, C–C, and C–C–O vibrations related to aromatic ring structures) were observed in ethanol-based extracts and UAE-E samples. Ultrasound treatment may promote the partial fragmentation of polymeric tannins in UAE-E samples due to cavitation effects, which facilitates the release of lower molecular weight phenolic compounds [36]. Water extraction produced a band at around 1047 cm^−1^, associated with C–O vibrations in alcohols and phenolic compounds. This band is also associated with carbohydrates and polymeric tannins found in pine bark. However, in the UAE-W extract, this band appeared less intense, suggesting that ultrasonic cavitation may modify or depolymerize phenolic polymers. Variations around 1360–1370 and 1200 cm^−1^ indicate that the phenolic fraction releases more easily via ultrasound than via conventional aqueous extraction.

The FTIR spectrum of the *β*-cyclodextrin extract exhibited distinctive features compared with the other extraction systems. Notably, the bands in the fingerprint region associated with phenolic structures were better preserved. A noticeable signal near ~1146 cm^−1^, attributed to C–O vibrations related to oligomeric phenolic structures, was observed in the *β*-cyclodextrin extract, suggesting effective recovery of condensed tannin components. Additionally, a strong band around ~1031 cm^−1^, associated with C–O, C–C, and aromatic ring vibrations, indicates the presence of phenolic compounds with preserved aromatic structures. In the 1280–1240 cm^−1^ region, which corresponds to phenolic C–O stretching and deformation vibrations, the *β*-cyclodextrin extract exhibited a relatively moderate but well-defined signal. This suggests a distinct distribution of phenolic functional groups compared to conventional solvent systems. These observations are consistent with the known ability of *β*-cyclodextrin to form inclusion complexes with aromatic molecules, which can enhance the solubility and stabilization of phenolic compounds during extraction.

#### 3.2.2. Matrix-Assisted Laser Desorption/Ionization Time-of-Flight Mass Spectrometry (MALDI-TOF-MS)

MALDI-TOF-MS is a highly sensitive and effective technique for detecting medium- and high-molecular-weight oligomers. This technique is particularly suitable for analyzing condensed tannins and other polymeric phenolic compounds [5]. Table 3 summarizes the main peaks detected in the spectra, along with their tentative assignments and the relative abundance of each peak for each extraction method. The MALDI-TOF-MS spectra for the different extraction systems are provided in the Supplementary Material (Figures S1a–f).

The MALDI-TOF spectra revealed peaks corresponding to oligomeric flavan-3-ol units typically associated with condensed tannins (proanthocyanidins). These oligomers were detected as sodium adducts [M + Na]^+^, which are commonly observed in MALDI analysis of phenolic oligomers. The identified peaks correspond primarily to procyanidin- and prodelphinidin-type structures, as well as fisetinidin-based oligomers, which are components of pine bark tannins [5,17]

Spectra revealed that most extracts exhibited abundant signals within the *m*/*z* 545–601 range, which corresponds to procyanidin dimers. These dimers are prevalent oligomeric units in *P. radiata* bark. Peaks at *m*/*z* 559 and 577 were abundant and were detected in several extraction systems, particularly with high relative abundances in ethanolic and water-based extracts. This suggests that these solvents effectively extract low-molecular-weight tannin oligomers.

Signals corresponding to trimers were observed in the *m*/*z* 843–905 range, including peaks tentatively assigned to fisetinidin trimers and procyanidin trimers. These oligomers were primarily detected in the alkaline extract, which suggests that alkaline conditions promote the release of higher-order tannin oligomers from the lignocellulosic matrix.

Procyanidin tetramers and prodelphinidin trimers were detected in the *m*/*z* 1057–1210 range. The *β*-cyclodextrin extract exhibited high signals at *m*/*z* 1158 and 1174, assigned to procyanidin tetramers. This indicates that the cyclodextrin-based extraction system may enhance the solubilization or stabilization of higher-molecular-weight tannins and the ability of *β*-cyclodextrin to form inclusion complexes with aromatic molecules, improving the extraction efficiency of polyphenolic compounds.

The spectra revealed high molecular weight oligomers, including procyanidin pentamers (*m*/*z* 1481), hexamers (*m*/*z* 1770), and heptamers (*m*/*z* 2058). These were mainly detected in water-based extracts (Water and UAE-W). Higher-degree polymerization suggests that aqueous extraction preserves polymeric tannin structures better than ethanol-based extraction systems.

The MALDI-TOF results confirm that *P. radiata* bark extracts are rich in condensed tannins, which are composed mainly of procyanidin-type oligomers with varying degrees of polymerization, consistent with the functional groups observed in the FTIR analysis. The observed oligomer distribution suggests that extraction polarity and matrix interactions strongly influence the recovery of tannins with different degrees of polymerization. Water-based and ultrasound-assisted water extractions favored the recovery of higher-molecular-weight tannins, while ethanol-based extraction predominantly recovered lower-molecular-weight oligomers. On the other hand, *β*-cyclodextrin extraction produced signals indicative of tetrameric procyanidins, suggesting that the stabilization and extraction of specific intermediate-molecular-weight phenolic structures were improved.

#### 3.2.3. Phenolic Profile by LC-LTQ-Orbitrap-MS

The phenolic composition of *P. radiata* bark extracts was studied using high-resolution LC-LTQ-Orbitrap mass spectrometry. Table 4 summarizes the main compounds tentatively identified in the extracts. A total of 16 phenolic compounds were tentatively identified, primarily phenolic acids, flavan-3-ols, and oligomeric proanthocyanidins, all of which have been previously reported in pine bark extracts [4,5]

Phenolic acids, including quinic acid, gallic acid, dihydroxybenzoic acid, and pro-tocatechuic acid, were detected in most extracts. Quinic acid (*m*/*z* 191.0558) was identified based on its fragmentation pattern, which included a precursor ion peak at *m*/*z* 191 and fragments at *m*/*z* 127 and 85 due to ring cleavage. Gallic acid (*m*/*z* 169.0142) was identified based on its exact mass and fragmentation pattern at *m*/*z* 125 due to loss of CO_2_. Dihydroxybenzoic acid (*m*/*z* 153.0193) and protocatechuic acid (*m*/*z* 153.0192) were identified based on their exact masses and fragmentation patterns, which were then compared to those in previous studies [18]. These phenolic acids were widely distributed among the extraction methods, indicating that they are extracted regardless of solvent polarity.

Two flavonoids, a flavonol and a flavanonol, were detected. Quercetin (*m*/*z* 301.0351) produced characteristic fragments at *m*/*z* 179 and 151, which are indicative of fragmentation via retro Diels–Alder (RDA) cleavage [18]. A taxifolin dimer (*m*/*z* 607.1086) was tentatively identified and produced prominent fragments at *m*/*z* 285, 177, and 125, consistent with a taxifolin fragmentation pattern. These flavonoids have been previously reported in *P. radiata* bark and contribute to its antioxidant properties [4,5].

The extracts also contained characteristic flavan-3-ol monomers, including (epi)catechin (*m*/*z* 289.0715) and (epi)gallocatechin (*m*/*z* 305.0665). These monomers are common building blocks of condensed tannins. (Epi)catechin produced characteristic fragmentation ions at *m*/*z* 245, which is attributed to the loss of CO_2_, as well as at *m*/*z* 205 and 179 due to cleavage of the A ring. This is typical of retro-Diels-Alder cleavage of the flavan-3-ol structure [18]. (Epi)gallocatechin produced characteristic fragments at *m*/*z* 179, 137, 125, and 109. These fragments are produced by heterocyclic ring fission (HRF) or retro-Diels-Alder (RDA) reactions [18]. These compounds were detected in nearly all extracts, confirming the presence of monomeric proanthocyanidin precursors in *P. radiata* bark.

Several oligomeric proanthocyanidins were also detected. These included procyanidin dimers (*m*/*z* 577), prodelphinidin dimers (*m*/*z* 593), procyanidin trimers (*m*/*z* 865), and a procyanidin tetramer that was detected as a doubly charged ion (*m*/*z* 576 [M−2H]^2−^). The compounds were identified based on their accurate masses and diagnostic MS/MS fragments, particularly ions at *m*/*z* 289, 407, and 125. These fragmentation patterns are characteristic of retro-Diels–Alder (RDA) reactions, heterocyclic ring fission (HRF), and quinone-methide cleavage (QM), which are commonly observed during proanthocyanidin fragmentation [18]. The detection of these oligomeric species aligns with MALDI-TOF-MS results, which also indicated the presence of condensed tannins with varying degrees of polymerization. Proanthocyanidins are a common type of oligomeric compound in *P. radiata* bark extracts [4].

The extraction method significantly impacts the diversity of phenolic compounds in *P. radiata* bark. The most diverse profile was yielded by alkaline extraction (16 compounds, as shown in Figure S2), followed by ultrasound-assisted ethanolic extraction (14) and conventional ethanolic extraction (13 compounds). Water extraction (11 compounds) yielded important flavan-3-ol and proanthocyanidin derivatives. The Orbitrap analysis confirmed that the extracts are composed of flavan-3-ols and oligomeric proanthocyanidins, as well as phenolic acids and flavonoids. This finding is supported by MALDI-TOF-MS, indicating the presence of high-molecular-weight tannin oligomers, offering a comprehensive molecular characterization of the extracts.

### 3.3. Thermal Stability of the Extract Obtained Under Optimal Conditions

#### 3.3.1. Thermogravimetric Analysis (TGA)

**Figure 10:**
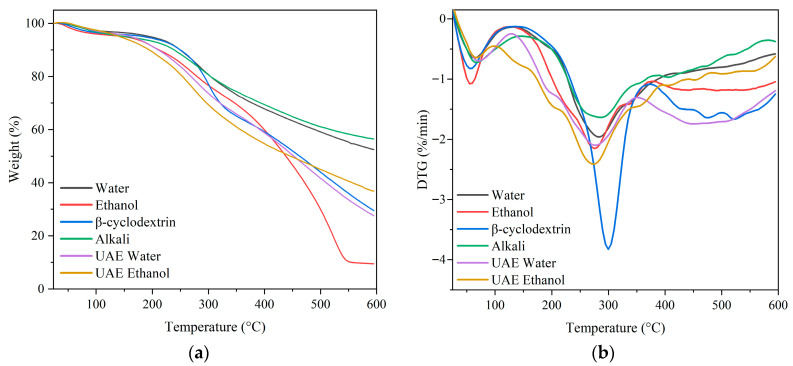
(**a**) Thermogravimetric analysis (TGA) and (**b**) differential thermogravimetric curves (DTG) of pine bark extracts obtained using different extraction methods.

Thermal stability is an important parameter for polyphenol-rich extracts, especially in industrial applications, where processing temperatures can impact the functional properties of bioactive compounds [37]. Figure 10 shows the thermogravimetric (TGA) and differential thermogravimetric (DTG) curves of pine bark extracts obtained using various extraction methods.

The thermal degradation patterns of the extracts revealed two main stages of weight loss. The initial stage occurred between 25 and 130 °C, with a minimum around 50–60 °C on the DTG curve, corresponding to the evaporation of adsorbed water and volatile compounds. This behavior is typical of plant-derived polyphenolic extracts due to the hygroscopic nature of phenolic compounds. The second stage, related to the thermal decomposition of organic constituents such as condensed tannins, phenolic structures, and carbohydrates, occurred between 150 and 400 °C [5,38]. Previous studies suggest that oligomeric tannins and some polysaccharides undergo thermal degradation above 200 °C. The DTG minimum was observed around 270–300 °C, which is consistent with previous findings for pine tannin extracts [4,5,37]

Alkaline extraction showed the highest thermal stability, followed by the conventional water extract. At 300 °C, both extracts retained about 80% of their initial mass, whereas the other systems retained 65–70%. These differences are more evident at higher temperatures. At 600 °C, alkaline and water extracts retained 50–55% of their initial mass, while the other systems retained only 10–35%. The higher thermal stability of the alkaline extract is attributed to the release of phenolic compounds from the bark matrix that change oligomeric tannin species distribution. The thermal behavior of tannin depends on its chemical structure and molecular weight. The breakdown of B-ring from tannin monomers, which leads to its cleavage, catalyzes further thermal degradation of the molecule [4]. These changes are agreed with the Orbitrap and MALDI-TOF analyses, which show a diverse profile of flavan-3-ols and proanthocyanidin oligomers.

The conventional ethanolic extract showed the lowest thermal stability at high temperatures, particularly above 450 °C, where a sharp mass loss was observed, reaching the lowest retained mass (about 10%) at around 550 °C. This behavior suggests the presence of thermally labile components, possibly associated with less polar, extractable fractions, such as lignin fragments, terpenoid compounds, sterols, and other lipophilic constituents commonly extracted with ethanol [37]. These results are also consistent with the MALDI results, where ethanol-based extraction predominantly recovered lower-molecular-weight oligomers. Similar behavior has been reported for methanolic extracts of *P. radiata* bark, containing tannin-insoluble fractions and other apolar compounds [37].

The *β*CD extract showed a pronounced DTG minimum near 300 °C, which can be attributed to the thermal decomposition of *β*-cyclodextrin. Pure *β*-cyclodextrin typically begins to decompose above 270 °C, with a major degradation event occurring around 290–320 °C, followed by carbonization processes at higher temperatures [14]. The shift and intensity of this decomposition peak in the extract suggest interactions between *β*-cyclodextrin and phenolic compounds, which may indicate the formation of inclusion complexes between *β*-cyclodextrin and aromatic polyphenols [14,39]. Overall, the TGA results indicate that the extraction method significantly influences the thermal stability of pine bark extracts, likely due to differences in phenolic composition, molecular weight distribution, and interactions between phenolic compounds and other matrix components.

#### 3.3.2. Differential Scanning Calorimetry (DSC)

**Figure 11:**
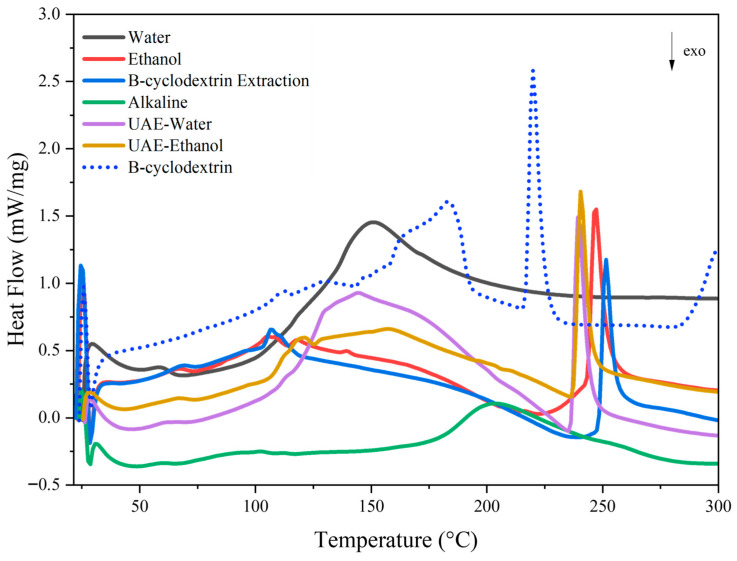
DSC thermograms of *P. radiata* bark extracts obtained using different methods (water, ethanol, *β*-cyclodextrin, alkaline, UAE-water, UAE-ethanol). The thermogram of pure *β*-cyclodextrin is included.

Differential scanning calorimetry (DSC) is commonly used to detect thermal events such as glass transitions, phase transitions, and decomposition processes by measuring the heat flow into or out of a sample as its temperature changes [40]. Figure 11 shows the DSC thermograms of the different pine bark extracts.

All samples exhibited endothermic bands associated with the transitions of phenolic compounds and dehydration processes. The initial endothermic signals appeared at 100 °C, 105 °C, 107 °C, and 120 °C for the alkaline, ethanol, *β-*cyclodextrin, and UAE-ethanol extracts, respectively. In contrast, the UAE-water and conventional water extracts displayed these signals at slightly higher temperatures, 144 °C and 150 °C, respectively. Mendoza-Wilson et al. [41] reported similar DSC behavior for catechin, a flavonoid monomer, showing two endothermic peaks around 100 and 150 °C, which were attributed to dehydration processes. Additionally, a broad endothermic band was observed in the water extract, with peaks around 150 and 203 °C. Endothermic signals above 150 °C may be linked to tannin decomposition, which involves breaking down aromatic structures and polysaccharide components, as well as the release of volatile products [42].

The thermogram of *β*-cyclodextrin is shown for comparison. This compound exhibited a broad endothermic region up to 200 °C with peaks at 113, 131, and 184 °C and a shoulder near 170 °C, followed by a more distinct transition at approximately 220 °C. An additional endothermic signal appeared at temperatures above 275 °C, corresponding to the thermal decomposition of *β*-cyclodextrin [14].

Previous studies have reported that the glass transition temperature (Tg) of *P. radiata* tannins typically occurs between 120 and 180 °C [4,37]. In the present study, Tg values ranging from 100 to 150 °C were observed, depending on the extraction method. The Tg of phenolic extracts can vary due to factors such as moisture content, molecular weight distribution, and the structural characteristics of the extracted tannins [4,42]. Alkaline extraction may reduce Tg due to modifications in the polymeric network and the presence of low-molecular-weight compounds, which is consistent with the oligomeric profile observed in the MALDI-TOF analysis.

### 3.4. Antimicrobial Activity of the Extract Obtained Under Optimal Conditions

The antibacterial activity of *P. radiata* extracts was evaluated against Gram-positive *S. aureus* and Gram-negative *E. coli*. Table 5 shows the antibacterial activity of the different *P. radiata* bark extracts.

Most extracts demonstrated inhibitory effects on *S. aureus*, whereas only UAE-E and UAE-W were effective against *E. coli*. This is attributed to the structural differences between Gram-positive and Gram-negative bacteria, with the latter having an outer membrane that limits antimicrobial penetration, increasing their resistance to plant extracts. Previous studies have similarly reported that Gram-positive bacteria are generally more susceptible to pine bark extracts than Gram-negative species [11,43,44]. Based on these results, the minimum inhibitory concentration (MIC) and minimum bactericidal concentration (MBC) were determined for the extracts that exhibited antibacterial activity (Table 6).

The extracts exhibited MIC values ranging from 0.04 to 1.40 mg/mL against *S. aureus.* However, the correlation between extract concentration and antibacterial effect was generally weak. In particular, the P-E and UAE-W extracts showed low R^2^ values, suggesting that the antibacterial response was not clearly dependent on extract concentration. A similar phenomenon was reported by Sánchez-Moya et al. [45], who observed increased *E. coli* proliferation with increasing concentrations of pine bark extract. This behavior highlights the complexity of antimicrobial responses in crude plant extracts.

The extracts P-W, UAE-E, and P-NaOH showed moderate antimicrobial activity against *S. aureus*, with MIC values ranging from 0.51 to 1.40 mg/mL. Notably, UAE-E exhibited activity against *E. coli*, with an MIC of 0.05 mg/mL. Previous studies have reported a wide range of MIC values for pine bark extracts. For example, Dönmez et al. [10] reported *S. aureus* MIC values ranging from 6.25 to 15 mg/mL using an aqueous-organic extraction method with *P. brutia* and *P. nigra* bark. In contrast, Barros et al. [11] reported lower MIC values of 0.01 mg/mL against *S. aureus* and 0.05 mg/mL against *E. coli* using aqueous extracts from *P. pinaster* bark, confirming the higher susceptibility of *S. aureus*. Studies specifically evaluating the antimicrobial activity of *P. radiata* bark extracts remain limited. Mun et al. [46] demonstrated that alkaline extraction of *P. radiata* bark using NaHCO_3_ significantly inhibited *S. aureus*.

The *β*-CD extract showed the most promising antimicrobial activity against *S. aureus*. This extract showed an MIC of 0.04 mg/mL and an MBC of 0.32 mg/mL, indicating that relatively low concentrations were sufficient to inhibit or eliminate bacterial growth. A stronger correlation between extract concentration and bacterial inhibition was also observed (R^2^ = 0.66), suggesting a more consistent antimicrobial effect.

These results indicate that *β*-cyclodextrin extraction may enhance the recovery and stabilization of bioactive phenolic compounds by improving solubility and stability for the encapsulation of bioactive compounds into *β*-CD, facilitating greater interaction with bacterial membranes and enhanced cell permeability [47].

Overall, these results suggest that the extraction method strongly influences the antibacterial potential of pine bark extracts. Specifically, *β*-cyclodextrin and UAE-E extraction showed the most promising activity against *S. aureus,* highlighting their potential for the development of antimicrobial biocomposites, biomaterials, or functional additives.

## 4. Conclusions

The RSM model optimized phenolic compound recovery, identifying temperature and solvent composition as key factors. The phenolic composition, thermal behavior, and antimicrobial activity of bark extracts from *P. radiata* varied depending on the extraction method. Alkaline extraction yielded the highest recovery rate (14.32%), and the β-CD-assisted system increased total phenolic content and antioxidant potential, particularly in the FRAP assay. Analyses confirmed the presence of flavan-3-ols and proanthocyanidin oligomers, including catechin structures. Thermal analyses revealed that alkaline extraction produced the most thermally stable extract, retaining approximately 50–55% of its initial mass at 600 °C. In contrast, the addition of *β*-cyclodextrin to the solvent enhanced the stability of the extracted phenolic compounds more effectively.

Antimicrobial assays revealed stronger activity against *S. aureus* than against *E. coli*. The *β*-cyclodextrin extract exhibited the remarkable antibacterial potency against *S. aureus*, with minimum inhibitory (MIC) and minimum bactericidal (MBC) concentrations of 0.04 and 0.32 mg/mL, respectively. Meanwhile, P-UAE-E exhibited activity against *E. coli* with an MIC of 0.05 mg/mL.

Overall, these results demonstrate that the extraction strategy significantly impacts the recovery and functionality of phenolic compounds from *P. radiata* bark. These findings highlight the potential of using *β*-cyclodextrin and ultrasound-assisted extraction to obtain bioactive extracts that can be used as natural antimicrobial agents and antioxidant additives. Future research should focus on incorporating these stabilized polyphenolic extracts into polymer matrices to develop active food packaging with antioxidant and antimicrobial properties.

## Figures and Tables

**Table 1:** Experimental variables involved in the study.

Variable	Definition	Value or Range	Units
Fixed	Solid-to-liquid ratio (S/L)	1/10	(*w*/*w*)
	Ethanol (% *v*/*v*)	80:20	(*v*/*v*)
Independent	Extraction temperature (°C)	20–80	T °C
	Extraction time (min)	20–100 (and 5–25) ^1^	min
	%NaOH and *β*CD	0.5, 1.0, 1.5% (db)	(*w*/*v*)
Dependent	Extraction yield	EY	(%)
	Total phenolic content	TPC	mg GAE/100 g db
	DPPH radical scavenging activity	DPPH	mg GAE/100 g db
	ABTS cation radical scavenging activity	ABTS	mg GAE/100 g db
	Ferric reducing antioxidant power	FRAP	mg GAE/100 g db

^1^ The ultrasonic extraction time ranged from 5 to 25 min.

**Table 2:** Summary of optimized *P. radiata* bark extraction parameters and main advantages.

Extraction Method	Temperature (°C)	Time (min)	Solvent/Agent	Key Advantages
Water	80	100	Water	Baseline performance for conventional extraction
Ethanol	80	100	80% Ethanol (*v*/*v*)	High antioxidant capacity (ABTS and DPPH)
UAE-Water	80	25	Water	Time-efficient and solvent-free process
UAE-Ethanol	80	25	80% Ethanol (*v*/*v*)	High yield and antioxidant capacity
Alkaline	80	100	1.5% NaOH (*w*/*w*)	Maximum overall extraction yield
*β*CD	80	100	*β*CD (*w*/*v*)	Maximum TPC and high antioxidant capacity (FRAP)

Water: conventional water extraction; Ethanol: conventional 80% (*v*/*v*) ethanol extraction; UAE Water: ultrasound-assisted water extraction; UAE Ethanol: ultrasound-assisted ethanol extraction; Alkali: NaOH-assisted extraction; *β*CD: *β* cyclodextrin-assisted extraction.

**Table 3:** MALDI-TOF-MS characterization of oligomeric proanthocyanidins in *P. radiata* bark extracts.

+Na^+^ (Exp.) (Da)	Tentative Identification	*β*CD (% R.A.)	Water (% R.A.)	UAE-W (% R.A.)	Ethanol (% R.A.)	UAE-E (% R.A.)	Alkaline (% R.A.)
545	Fisetinidin dimer	3	ND	ND	ND	ND	ND
559	Procyanidin dimer	12	77	77	100	42	ND
561	Procyanidin dimer	3	ND	15	ND	ND	53
577	Procyanidin dimer	9	77	100	77	39	ND
579	Procyanidin dimer	3	60	38	ND	ND	ND
601	Procyanidin dimer	2	24	ND	ND	ND	100
747	Prodelphinidin dimer	ND	60	ND	ND	ND	ND
769	Prodelphinidin dimer	ND	32	ND	ND	ND	ND
843	Fisetinidin trimer	ND	ND	ND	ND	12	ND
845	Fisetinidin trimer	2	ND	17	35	24	ND
889	Procyanidin trimer	ND	24	ND	ND	ND	70
903	Procyanidin trimer	ND	14	ND	ND	ND	21
905	Procyanidin trimer	4	100	33	ND	ND	29
1057	Procyanidin tetramer	ND	18	ND	ND	ND	ND
1158	Procyanidin tetramer	42	ND	ND	ND	ND	ND
1174	Procyanidin tetramer	100	ND	ND	ND	ND	ND
1177	Prodelphinidin trimer	ND	9	ND	ND	ND	41
1191	Prodelphinidin trimer	5	9	10	ND	ND	20
1193	Procyanidin tetramer	ND	48	42	ND	ND	20
1210	Prodelphinidin trimer	1	14	10	ND	ND	ND
1481	Procyanidin pentamer	ND	27	34	ND	ND	ND
1770	Procyanidin hexamer	3	15	26	ND	ND	ND
2058	Procyanidin heptamer	2	7	16	ND	ND	ND

Exp.: Experimental; *β*CD: *β*-cyclodextrin extraction; UAE-W: ultrasound-assisted water extraction; UAE-E: ultrasound-assisted ethanol extraction; ND: not detected; R.A.: relative abundance.

**Table 4:** Identification of phenolic compounds in the *P. radiata* bark extracts by LC-ESI-LTQ-Orbitrap-MS in negative mode.

Tentative Identification	Rt (min)	ESI (*m*/*z*)	MS/MS ESI (−) *m*/*z*	Alkaline	*β*CD	UAE-E	Ethanol	UAE-W	Water
Quinic acid	1.50	191.0558	191.0559, 127.0401, 85.0295	✓	✓	✓	✓	ND	✓
Gallic acid	4.30	169.0142	125.0240	✓	✓	✓	✓	✓	✓
Dihydroxybenzoic acid	7.66	153.0193	153.0189, 123.0449	✓	✓	✓	✓	✓	✓
Protocatechuic acid	10.21	153.0192	109.0293	✓	✓	✓	✓	✓	✓
(Epi)gallocatechin	11.05	305.0665	179.0347, 137.0243, 125.0240, 109.0290	✓	✓	✓	✓	✓	ND
Prodelphinidin dimer	12.08	593.1302	407.0764, 289.0712, 245.0813, 125.0243	✓	ND	✓	ND	✓	✓
Procyanidin dimer B	14.02	577.1348	407.0766, 289.0718, 125.0244	✓	✓	✓	✓	✓	ND
Procyanidin dimer B	14.55	577.1346	407.0767, 289.0720, 125.0244	✓	✓	✓	✓	✓	✓
(Epi)catechin	15.06	289.0715	245.0817, 205.0504, 179.0350	✓	✓	✓	✓	✓	✓
Procyanidin trimer	15.37	865.1989	407.0758, 289.0715, 245.0453, 125.0242	✓	✓	ND	ND	ND	ND
Procyanidin trimer	15.51	865.1984	407.0759, 289.0713, 245.0453, 125.0242	✓	ND	✓	✓	✓	✓
Procyanidin tetramer *	16.41	576.1276	407.0767, 289.0715, 125.0243	✓	ND	ND	ND	ND	ND
Procyanidin dimer B	17.04	577.1350	407.0764, 289.0717, 245.0813, 205.0503, 179.0347, 125.0243	✓	ND	✓	✓	✓	ND
Procyanidin dimer B	17.78	577.1351	407.0765, 289.0716, 245.0813, 205.0498, 179.0355, 125.0243	✓	ND	✓	✓	✓	✓
Taxifolin dimer	18.17	607.1086	285.0406, 177.0190, 125.0244,	✓	✓	✓	✓	✓	✓
Quercetin	18.51	301.0351	178.9986, 151.0036	✓	ND	✓	✓	✓	✓

Rt, retention time; ND, not detected; UAE-E, ultrasound-assisted ethanol extraction; UAE-W, ultrasound-assisted water extraction; *β*CD, *β*-cyclodextrin extraction. ✓ indicates presence of the compound. * Procyanidin tetramer was detected as a doubly charged ion.

**Table 5:** Antibacterial activity of the different *P. radiata* bark extracts against *E. coli* and *S. aureus* determined by the agar diffusion method.

Extracts	Halo Diameters (mm)	
	*E. coli*	*S. aureus*
Ethanol	ND	11.6 ± 0.2 ^ac^
Alkaline	ND	11.3 ± 0.1 ^a^
Water	ND	11.2 ± 0.9 ^ab^
*β*-cyclodextrin	ND	10.6 ± 0.6 ^b^
UAE-E	11.6 ± 0.7 ^aA^	13.5 ± 1.2 ^dB^
UAE-W	11.5 ± 1.8 ^aA^	11.4 ± 0.4 ^abcA^

Lowercase letters indicate significant differences within rows, while uppercase letters indicate significant differences between bacterial strains (*p* < 0.05). ND: no detectable antimicrobial activity. Original disk diameter: 10 mm.

**Table 6:** Minimum inhibitory concentration (MIC) and minimum bactericidal concentration (MBC) of *P. radiata* bark extracts against *S. aureus* and *E. coli.*

Extracts	*S. aureus*			*E. coli*		
	MIC	MBC	*R* ^2^ **	MIC	MBC	*R*^2^ **
	(mg/mL)	(mg/mL)		(mg/mL)	(mg/mL)	
UAE-W	w.e. *	w.e. *	0.298	w.e. *	w.e. *	0.032
Ethanol	w.e. *	w.e. *	0.136	w.e. *	w.e. *	-
Water	0.51	3.98	0.055	w.e. *	w.e. *	-
*β*-cyclodextrin	0.04	0.32	0.660	w.e. *	w.e. *	-
UAE-E	1.40	11.25	0.005	0.05	3.97	0.005
NAOH	1.08	9.52	0.001	w.e. *	w.e. *	-

* w.e.: without effect; ** Correlation factor.

## Data Availability

The original contributions presented in this study are included in the article/Supplementary Materials. Further inquiries can be directed to the corresponding author.

## Supplementary Materials

The following supporting information can be downloaded at: https://www.mdpi.com/article/10.3390/antiox15050565/s1, Table S1: ANOVA results for the response surface models of *P. radiata* bark extraction; Figure S1: MALDI-TOF-MS spectra of *P. radiata* bark extracts. Figure S2. Total Ion Chromatogram (TIC) of the *P. radiata* bark alkaline extract.
