# Supplementary material for: Optimization of Emerging Extraction Techniques for Phenolic Compounds from Pinus radiata Bark: Antioxidant, Thermal Stability and Antibacterial Properties
Source: Antioxidants (Basel). 2026 Apr 29;15(5):565. doi: 10.3390/antiox15050565 (PMC13203268; doi:10.3390/antiox15050565)
Supplement: Supplementary file 1 [file antioxidants-15-00565-s001.zip › antioxidants-4233186-supplementary.pdf]

antioxidants

MDPI

Supplementary Materials

Table S1. ANOVA results for the response surface models of *P. radiata* bark extraction

Dependen-dant variable	Extraction Method	Model (p-value)	Temperature (p-value)	Time (p-value)	Concentra-tion (p-value)	Lack of Fit (p-value)
Yield	Water	0.0030	0.0026	0.0020	-	0.3234
	Ethanol	< 0.0001	< 0.0001	0.0005	-	0.0711
	UAE-Water	0.0017	0.0031	0.0064	-	0.6925
	UAE-Ethanol	0.0015	< 0.0001	0.0742	-	0.0671
	Alkali	< 0.0001	< 0.0001	0.0468	< 0.0001	0.8539
	βCD	< 0.0001	< 0.0001	< 0.0001	0.0787	0.3709
TPC (Folin-Ciocal-teu)	Water	0.0119	0.0251	0.0204	-	0.5678
	Ethanol	0.0063	0.0020	0.5273	-	0.1257
	UAE-Water	0.0070	0.0028	0.2419	-	0.6465
	UAE-Ethanol	0.0489	0.0253	0.2678	-	0.6466
	Alkali	0.0120	0.0057	0.0374	0.0156	0.1175
	βCD	0.0022	0.0288	0.0051	0.1112	0.2972
DPPH	Water	< 0.0001	< 0.0001	0.4540	-	0.2158
	Ethanol	0.0010	0.0009	0.0166	-	0.3248
	UAE-Water	0.0046	0.0026	0.1017	-	0.3747
	UAE-Ethanol	0.0273	0.0120	0.3066	-	0.9384
	Alkali	0.0002	0.0133	0.0003	0.0153	0.4737
	βCD	0.0202	0.0030	0.0471	0.1852	0.6481
ABTS	Water	0.0006	0.0002	0.5963	-	0.8720
	Ethanol	0.0015	0.0007	0.0994	-	0.8505
	UAE-Water	0.0039	0.0011	0.8972	-	0.5993
	UAE-Ethanol	< 0.0001	< 0.0001	0.0021	-	0.3362
	Alkali	0.0137	0.0172	0.5921	0.0145	0.8994
	βCD	0.0021	0.0002	0.6132	0.8788	0.5834
FRAP	Water	0.0139	0.0004	0.2699	-	0.1221
	Ethanol	0.0230	0.0079	0.5764	-	0.7725
	UAE-Water	0.0236	0.0379	0.0402	-	0.8789
	UAE-Ethanol	0.0075	0.0142	0.0193	-	0.6345
	Alkali	0.0012	0.0002	0.3498	0.3454	0.9173
	βCD	0.0197	0.0105	0.0763	0.2495	0.2410

The methods were: water (conventional water extraction), ethanol (conventional 80% v/v ethanol extraction), ultrasound-assisted water extraction (UAE-water), ultrasound-assisted ethanol extraction (UAE-ethanol), alkali (NaOH-assisted extraction), and β-cyclodextrin (β-CD)-assisted extraction. One-way ANOVA followed by Tukey's HSD post hoc test ($p < 0.05$) was used to determine significant differences between the maximum values of each method.

Figure S1. MALDI-TOF-MS spectra of *P. radiata* bark extracts.

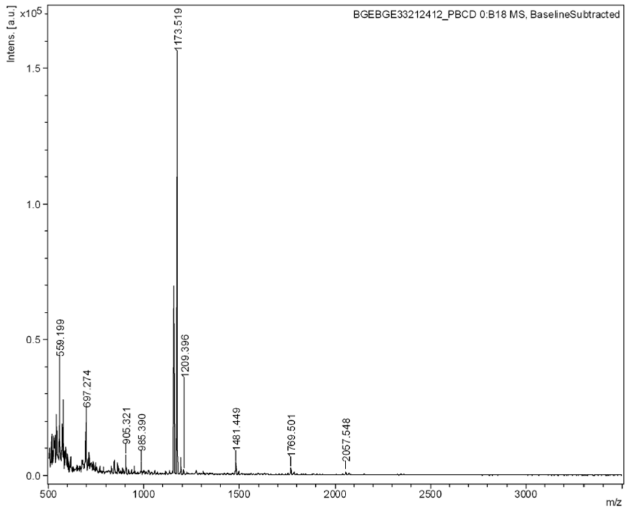

(a) *β*-cyclodextrin

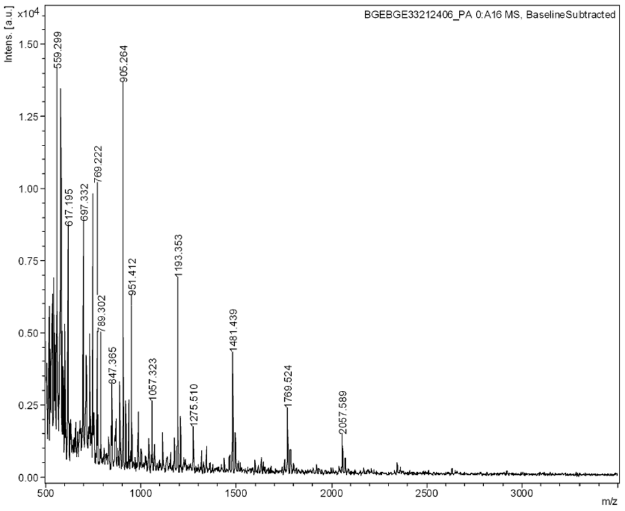

(b) Water

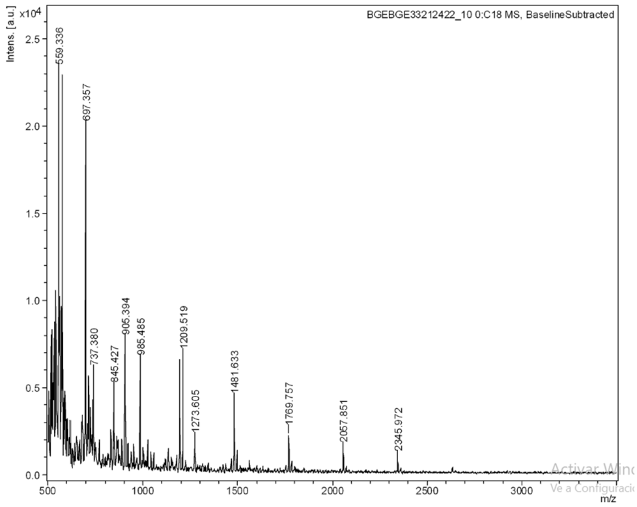

(c) UAE-W

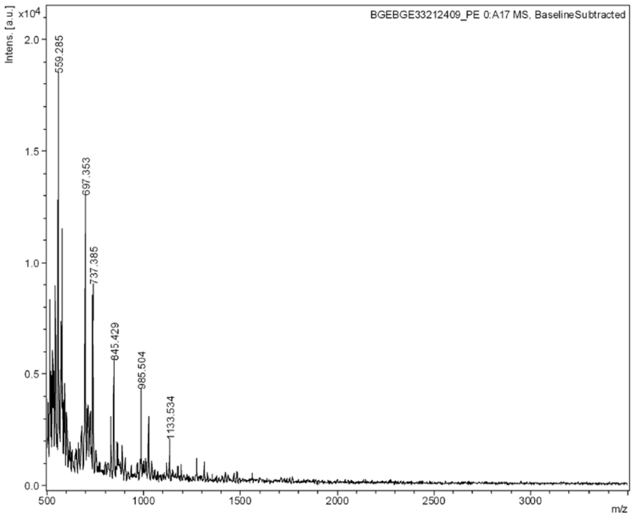

(d) Ethanol

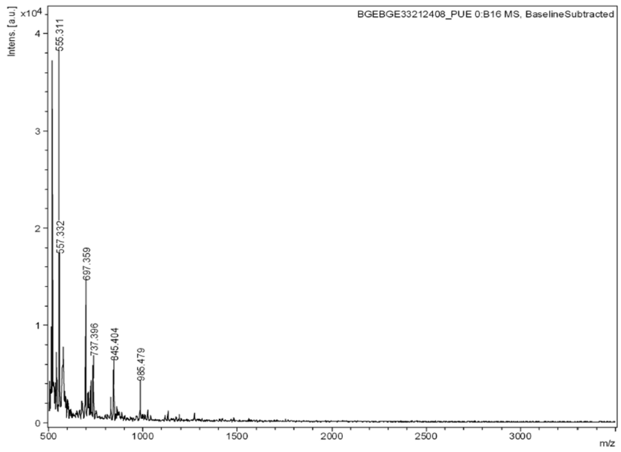

(e) UAE-E

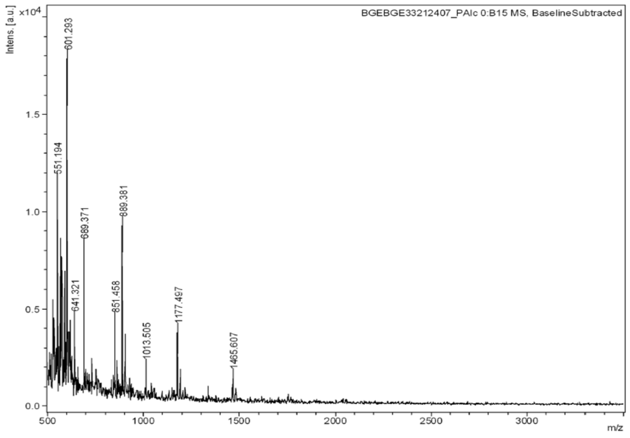

(f) Alkaline

Experimental mass spectra obtained by MALDI-TOF-MS. (a) β-cyclodextrin: βCD-assisted extraction; (b) Water: conventional water extraction; (c) UAE-W: ultrasound-assisted water extraction; (d) Ethanol: conventional 80% (v/v) ethanol extraction; (e) UAE-E: ultrasound-assisted ethanol extraction; : (f) Alkaline-assisted extraction.

Figure S2. Total Ion Chromatogram (TIC) of the *P. radiata* bark alkaline extract.

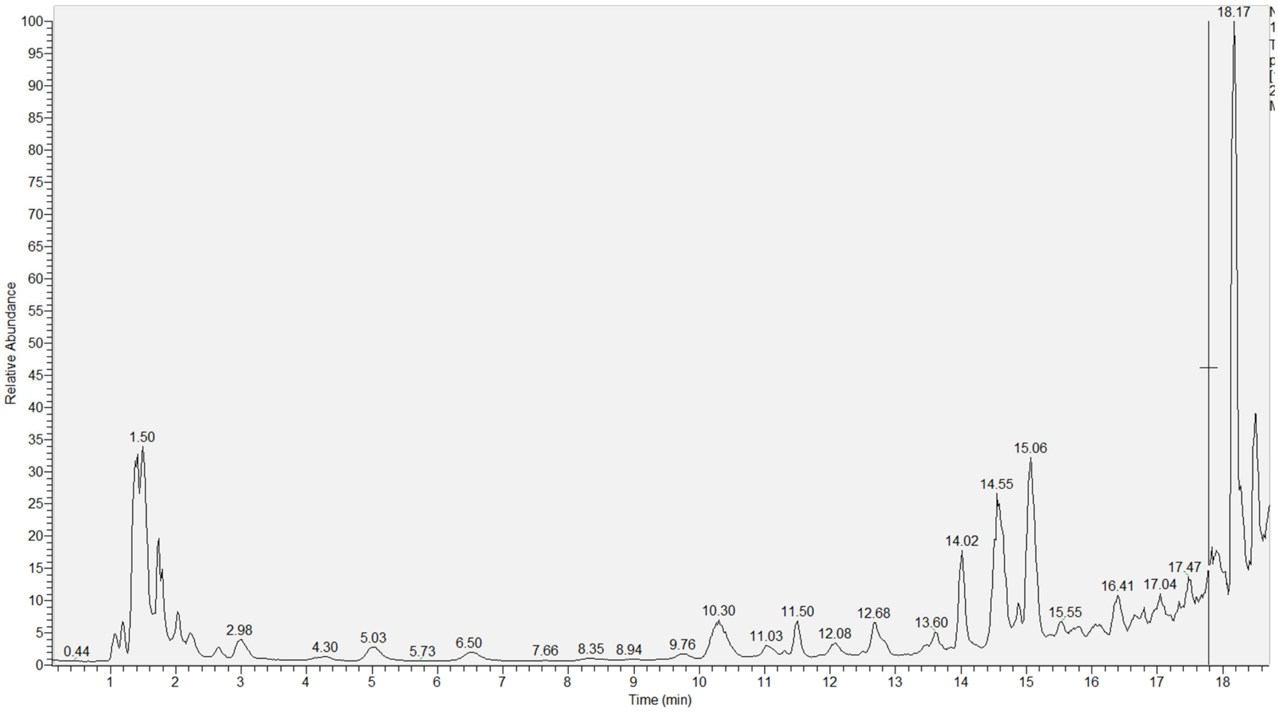

Total Ion Chromatogram (TIC) of *the P. radiata* bark alkaline extract, obtained by LC-ESI-LTQ-Orbitrap-MS in negative ion mode.
